# Long-term cardiovascular consequences of adolescent anorexia nervosa

**DOI:** 10.1038/s41390-023-02521-5

**Published:** 2023-02-15

**Authors:** Gabriella A. C. Springall, Michelle Caughey, Diana Zannino, Kypros Kyprianou, Jonathan P. Mynard, Subashini Rudolph, Jeanie Cheong, Michele Yeo, Michael M. H. Cheung

**Affiliations:** 1 Department of Paediatrics, University of Melbourne, Parkville, VIC Australia; 2 Murdoch Children’s Research Institute, Parkville, VIC Australia; 3 Department of Adolescent Medicine, Monash Children’s Hospital, Clayton, VIC Australia; 4 Clinical Epidemiology and Biostatistics Unit, Murdoch Children’s Research Institute, Parkville, VIC Australia; 5 Monash University, Clayton, VIC Australia; 6 Department of Biomedical Engineering, University of Melbourne, Parkville, VIC Australia; 7 Department of Paediatric Medicine, Austin Health, Heidelberg, VIC Australia; 8 Department of Obstetrics and Gynaecology, University of Melbourne, Parkville, VIC Australia; 9 Neonatal Services, Royal Women’s Hospital, Parkville, VIC Australia; 10 Department of Adolescent Medicine, Royal Children’s Hospital, Parkville, VIC Australia; 11 Department of Cardiology, Royal Children’s Hospital, Parkville, VIC Australia

## Abstract

**Background:**

Anorexia nervosa (AN) is associated with maladaptive cardiovascular changes. This study investigated whether individuals who recovered from AN during adolescence experience long-term cardiovascular risk in early adulthood.

**Methods:**

Former AN patients discharged from the Royal Children’s and Monash Children’s Hospital Eating Disorder Services in Melbourne, Australia underwent cardiovascular testing. Measurements were performed using an oscillometric device for blood pressure and pulse wave velocity, ultrasound for carotid wall structure/function, resting electrocardiogram for heart-rate variability, and the EndoPat 2000 (Itamar) system for endothelial function. Patient measures were compared to healthy controls and/or normal thresholds.

**Results:**

Ninety-one percent of the former AN patients (*N* = 22) and controls (*N* = 66) were female, aged approximately 25 years, with a healthy body mass index. The mean time interval from AN recovery to participation was 7.4 years. Pulse wave velocity was lower in the former AN patients than controls. Carotid intima–media thickness was not different; however, carotid distensibility and compliance were lower, and the elastic modulus higher in the former AN patients. Greater vagal tone was observed and endothelial dysfunction was evident in 46% of the former patients.

**Conclusions:**

Young adults who recovered from adolescent AN exhibit persistent cardiovascular adaptations. Routine cardiovascular monitoring could manage potential disease risk.

**Impact:**

- Cardiovascular complications are common in patients with anorexia nervosa (AN) and population studies have revealed that developmental adaptations in response to undernutrition have long-term consequences for cardiovascular health.
- In this study of young adults treated for AN during adolescence, there was evidence of increased carotid artery stiffness, reduced aortic stiffness, vagal hyperactivity, and endothelial dysfunction in early adulthood when compared to healthy controls.
- It is important to consider the cardiovascular health of patients with AN beyond achieving medical stability.
- Interventions that monitor cardiovascular health could minimise the burden of future cardiovascular disease.

## Introduction

Anorexia nervosa (AN) has a high rate of mortality among psychiatric disorders.^1,2^ The leading cause of hospitalisation are cardiovascular complications which occur in up to 80% of cases and account for at least one third of all patient deaths.^3,4^ Profound structural, electrophysiological, and functional cardiovascular adaptations arise due to the disease.

Changes in cardiac size and function during the acute phase of illness are well described.^4–6^ Changes in heart rhythm arise from a shift in autonomic regulation towards parasympathetic dominance. These adaptations have been associated with sudden cardiac death.^4,7^ Functional impairment of the cardiovascular system occurs due to the structural and electrophysiological adaptations. Orthostatic hypotension and a lack of normal circadian variations in blood pressure are common, along with impaired endothelial function.^8,9^ Many of these cardiac complications are thought to resolve with refeeding and weight gain. Whether the myocardial fibrosis in patients with AN will resolve over time is less clear.^9,10^ This affects contractility and may contribute to increased cardiovascular mortality.

Furthermore, changes in vascular properties in patients with AN after weight restoration have not been reported in detail. Studies of populations exposed to malnutrition have revealed alterations in cardiovascular structure and function in later life. For example, early onset of coronary artery disease has been reported among survivors of famine.^11,12^ An increased incidence of atherosclerosis and ischaemic heart disease has been observed among former prisoners of war.^13,14^ Adult survivors of severe acute malnutrition in infancy have also exhibited altered cardiac structure accompanied by elevated peripheral resistance.^15^ Although the types of malnutrition in this study population were varied, there were reductions in cardiac output, stroke volume, and outflow tract diameter. Such studies indicate that periods of significant malnutrition in early life have long-term consequences for cardiovascular health.

To date, most studies assessing cardiovascular risk factors in AN have been conducted in patients currently undergoing treatment. The long-term cardiovascular prognosis post-recovery is unknown and indeed most patients are discharged from clinical services. Using well-established assessment techniques employed widely in children and adults,^16^ the present study aimed to determine the presence of long-term cardiovascular changes in individuals who were malnourished during adolescence due to an eating disorder.

## Methods

### Setting

This study took place at the specialist Eating Disorder programmes operating at the Royal Children’s Hospital (RCH) Melbourne and Monash Children’s Hospital. These multidisciplinary services provide inpatient and outpatient care to patients under 18 years of age who are diagnosed with an eating disorder according to the Diagnostic and Statistical Manual of Mental Disorders.^17^

### Study sample

The study population comprised of a sample of former patients who were treated for AN and clinically discharged from the Eating Disorder Services between June 2008 and July 2016. Of these patients, 36 had previously undergone psychological assessment^18^ and expressed interest in the cardiovascular component of the research. Patient commitment to exercise was comparable to the general population according to the Commitment to Exercise Scale. Twenty-two participants were successfully recruited from the potential pool of 36 former AN patients. Nine patients could not be contacted and the remaining five declined participation due to other time commitments or no longer residing in Melbourne. To be included, participants had been discharged (discharge requires an Eating Disorder Examination (EDE) Global Score within 1 SD of community norms and a body mass index (BMI) ≥ 95% of the median for age and sex) from the Eating Disorder Service ≥5 years ago, were aged >18 years, exhibited a current EDE Global Sore within 1 SD of community norms, and possessed a BMI ≥ 18.5 kg/m^2^. Additional historical information was obtained from patients’ medical records, including the duration, severity, and age at AN presentation, as well as the presence of any physical or mental health comorbidities.

Healthy controls did not have a previous diagnosis of an eating disorder themselves or among their first-degree relatives and did not have any known chronic disease. Controls were drawn from the Victorian Infants Collaborative Study (VICS),^19^ a longitudinal study of extremely preterm infants that included a term-born control group. Controls were matched to former AN patients by age and sex with a ratio of 3:1. Cardiovascular data in the AN group was obtained prospectively and employed the same protocols for collection of data as the VICS control group.

A sample size of 88 participants (22 former AN and 66 control) would enable differences of at least 0.7 SD to be detected with 80% power using a two-sided type I error of 5%.

### Study procedures

#### Carotid intima–media thickness and elasticity

Participants underwent non-invasive cardiovascular assessment by a trained researcher at the RCH vascular research laboratory. Testing was conducted in the morning, in the absence of caffeine intake and after fasting for at least 3 h. While lying in the supine position with the head turned 45° to the left, ultrasound of the right carotid artery was performed with a GE Vivid ultrasound machine (Vivid i BT06, 10–15 MHz linear array probe; GE Healthcare). Image analysis was performed offline using the Carotid Analyser Software for Research (Medical Imaging Applications, Coralville, IA). Digital callipers were subsequently used to measure carotid intima–media thickness (IMT) of the far wall of the common carotid artery 5–10 mm from the carotid bulb at end-diastole (R-wave of ECG); with intima–media thickening a proven pre-clinical marker of atherosclerotic risk.^20^ Maximal and minimal vessel diameters were measured to examine vessel distensibility. Brachial blood pressure was used to calculate vessel compliance and the incremental elastic modulus. These measures indicate carotid arterial stiffness, and thereby the extent of vascular aging or atherosclerosis.^20^

#### Pulse wave analysis and carotid–femoral pulse wave velocity

Central and peripheral blood pressure were measured on the non-dominant arm using a SphygmoCor XCEL oscillometric device (Atcor Medical, Sydney, Australia). The peripheral measurement was taken directly and central pressure estimated from this using in-built functions. While more difficult to measure, there is increasing evidence of the strength of this parameter as a measure of cardiovascular risk due to varied augmentation of blood pressure in peripheral vessels.^21^ Measurements were also made of the carotid–femoral pulse wave velocity as a surrogate of aortic stiffness. This was performed with a hand-held tonometer at the carotid pulse and a proximal thigh cuff. The distance from the carotid pulse to the suprasternal notch, from the suprasternal notch to the right femoral pulse, and from the femoral pulse to the top of the thigh cuff were measured with a tape measure to record the distance travelled by the waveforms.^22^ The time difference between the foot of the two waveforms was used for pulse wave velocity calculation. All blood pressure and pulse wave velocity measurements were performed in triplicate and the average calculated for each participant.

#### Heart-rate variability (HRV)

A three-lead electrocardiogram was recorded for 15 min using a PowerLab system operating with the LabChart 8 software (ADInstruments, UK). Resting heart rate and heart rate variation during normal breathing were measured at a sampling rate of 1 k/s. As a measure of autonomic nervous system function, short-term HRV analysis was conducted in the LabChart HRV add-on version 2.0 using the Lomb Periodogram nonparametric method for power spectral density estimation. Analysis parameters included a window size of 200 sampling points with 50% overlap, a model order of 18, percentage of successive RR intervals (pRR) threshold of 50 ms, and standard deviation of the set of averaged RR intervals averaging over 300 s. Time and frequency domain measures were obtained in accordance with guidelines developed by the Task Force of the European Society of Cardiology and the North American Society of Pacing and Electrophysiology.^23^

#### Endothelial function

Assessment of the endothelial-dependent flow-mediated vasodilation of arteries is a well-established measure of arterial vessel health.^24,25^ In this study, we captured a beat-to-beat plethysmographic recording of the finger arterial pulse wave amplitude in response to reactive hyperaemia using the EndoPAT 2000 (Itamar Medical, Israel) system. Pneumatic finger probes were placed on the index fingers of each hand and a brachial blood pressure cuff was placed on the dominant arm. A baseline recording was obtained for 5 min at rest followed by arterial occlusion of the dominant arm via cuff inflation to supra-systolic pressure for 5 min. A 5 min recording measured reactive hyperaemia following blood pressure cuff deflation. The magnitude of flow-mediated hyperaemia was calculated as the ratio between baseline and post-occlusion pulse wave amplitude (reactive hyperaemic index (RHI)), corrected for systemic changes measured in the non-occluded arm. The VICS cohort did not undergo this assessment of endothelial function.^19^ A threshold RHI <1.67 is considered abnormal in adults.^26^

### Statistical analysis

General descriptive statistics of the former AN and control samples were calculated. Clinical characteristics of the former AN patients at the time of their diagnosis were also summarised. The mean and standard deviation (SD) were reported for physically obtained cardiovascular data that were normally distributed. For data that were not normally distributed, median and interquartile range (IQR) were reported. The mean difference and corresponding 95% confidence interval (CI) between the controls and former AN patients with respect to each of the measures of carotid IMT, blood pressure, pulse wave velocity, and HRV were estimated using multivariable linear regression with group as the variable of interest, adjusted for age and sex. Quantile regression was similarly used to estimate median differences and corresponding 95% CIs for skewed variables. In addition, the RHI of former AN patients was compared to normal reference values and the percentage of the sample with abnormal readings reported. Given the number of comparisons performed in our study, we considered *p* ≤ 0.005 to indicate strong evidence of a difference and 0.005 < *p* < 0.05 to indicate moderate evidence of a difference. All statistical analyses were performed using the R software (version 4.0.1, R Foundation, Vienna, Austria).

## Results

### Population

The former AN patients (*N* = 22) and the control group (*N* = 66) were aged approximately 25 years (24.5 (2.7) and 24.9 (0.91), for former AN patients and controls, respectively) and had a BMI within the healthy range (22.36 (2.61) and 23.76 (2.24), *p* = 0.017, for former AN patients and controls, respectively). The majority (91%) of former AN patients were female. The time since discharge from their respective Eating Disorder Services to study participation ranged from 5 to 10 years with a mean (SD) interval of 7.4 (2.1) years. Two (9%) of the former AN patients smoked and 15% of the female patients reported the presence of amenorrhoea.

Table 1 presents characteristics from the former AN patients initial clinical assessment and diagnosis. Mean age and BMI at diagnosis was approximately 16 years and 16.28 kg/m^2^, respectively. This indicates a moderately malnourished patient cohort.^17^ The patients had lost an average of 26% (ranging from 10 to 40%) of their body weight over a period of 4–24 months. Medical instability was prevalent, with more than half the patients experiencing bradycardia, and more than a third experiencing postural hypotension. Anxiety affected approximately one quarter of the patients; while other mental health and medical comorbidities were less frequently reported.

**Table 1:** Characteristics of the AN study sample at diagnosis.

	Sample data (*N* = 22)
Initial clinical measure	
Age at diagnosis (years), mean (SD)	15.8 (1.4)
BMI at diagnosis (kg/m^2^), mean (SD)	16.28 (1.87)
Quantity of weight loss (%), mean (SD)	26 (11)
ED duration (months), mean (SD)	15.2 (11.3)
Patients admitted (%)	73
Length of stay (days), mean (SD)	18.4 (21.6)
Bradycardia (%)	55
Postural hypotension (%)	32
Required electrolyte supplementation (%)	23
Required nasogastric feeding (%)	14
Purging (%)	23
Mental health comorbidities	
Depression (%)	23
Anxiety (%)	18
Self-harm ideation (%)	18
Suicidal ideation (%)	9
Medical comorbidities	
Asthma (%)	18
Chronic fatigue syndrome (%)	5

### Pulse wave analysis and velocity

**Fig. 1:**
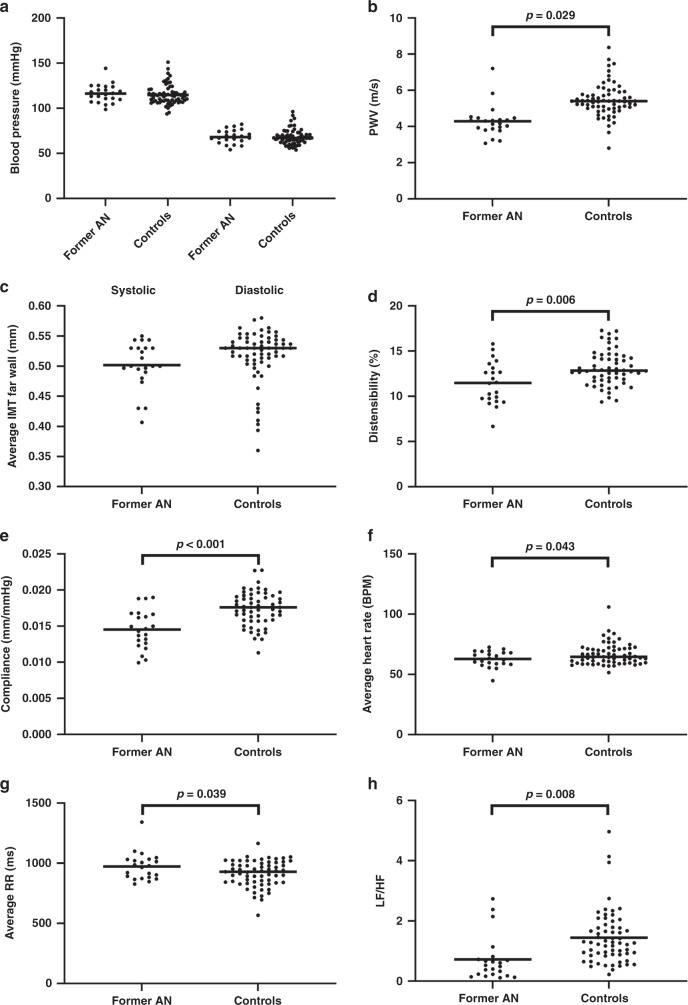
Primary cardiovascular measures of former AN patients and controls. **a** Blood pressure, **b** pulse wave velocity (PWV), **c** average carotid intima-media thickness (IMT) of the far wall, **d** carotid distensibility, **e** carotid compliance, **f** average heart rate, **g** average RR interval, and **h** low-frequency/high-frequency power (LF/HF).

There was no evidence of a difference in peripheral or central blood pressure between the two groups (Table 2 and Fig. 1a). There was moderate evidence of decreased aortic stiffness with former AN patients exhibiting lower pulse wave velocity than the control group (adjusted mean difference −1.1, 95% CI −1.5 to −0.9, *p* = 0.029) (Fig. 1b). Measures of pulse augmentation (indirect measures of arterial stiffness obtained from looking at the central pressure waveform) were similar in the former AN patients and controls.

**Table 2:** Pulse wave analysis and velocity of former AN patients and controls.

	Former AN (*N* = 22)	Controls (*N* = 66)	Estimated mean difference (95% CI)
Blood pressure measures, mmHg			
Systolic	116.3 (10.1)	114.8 (10.7)	0.8 (−10.2, 11.8)
Diastolic	68.6 (7.6)	68.0 (8.5)	−0.9 (−10.1, 8.2)
Pulse pressure	46.9 (6.7)	46.7 (6.2)	1.72 (−4.4, 7.8)
Mean arterial pressure	82.5 (9.2)	81.3 (10.1)	0.2 (−10.7, 11.1)
Central systolic	102.3 (9.4)	100.1 (9.6)	1.8 (−8.2, 11.8)
Central diastolic	69.6 (7.7)	68.9 (9.1)	−1.1 (−10.9, 8.7)
Central pulse pressure	33.4 (5.5)	31.1 (4.7)	2.9 (−1.5, 7.5)
Augmented pressure	3.5 (4.8)	2.1 (3.5)	2.6 (−1.1, 6.3)
Pulse wave analysis, %			
Augmentation index	9.6 (13.5)	5.9 (10.2)	7.5 (−3.2, 18.2)
Augmentation index @ 75^a^	4.1 (15.5)	1.8 (10.7)	6.5 (−5.2, 18.3)
Other measures			
Heart rate, BPM	64 (10)	66 (11)	−1 (−14, 11)
Pulse wave velocity, m/s	4.3 (0.9)^b^	5.3 (0.8)	−1.1 (−1.5, −0.9)

Data are mean (SD).

^a^Heart rate-corrected augmentation index.

^b^*p* < 0.05.

### Carotid artery properties

There was no evidence of a difference in the carotid IMT or carotid artery diameters between former AN patients and controls (Table 3 and Fig. 1c). There was moderate-strong evidence of former AN patients having stiffer carotid vessels; exhibiting lower distensibility (adjusted mean difference −1.63, 95% CI −2.65 to −0.61, *p* = 0.006), lower compliance (adjusted mean difference −1.63, 95% CI −1.83 to −1.44, *p* < 0.001) (Fig. 1d, e), and a higher incremental elastic modulus (adjusted mean difference 214.69, 95% CI 131.2 to 298.18, *p* < 0.001) than controls.

**Table 3:** Carotid ultrasound of former AN patients and controls.

	Former AN (*N* = 22)	Controls (*N* = 66)	Estimated mean difference (95% CI)
Arterial structure, mm			
Average IMT far wall	0.50 (0.04)	0.52 (0.04)	−0.20 (−2.50, 2.09)
Maximum carotid lumen diameter	5.92 (0.42)	5.87 (0.41)	−0.13 (−0.54, 0.28)
Minimum carotid lumen diameter	5.20 (0.36)	5.06 (0.34)	−0.07 (−0.41, 0.26)
Arterial stiffness			
Distensibility, %	11.48 (2.33)^a^	13.11 (1.92)	−1.63 (−2.65, −0.61)
Compliance, mm/mmHg × 10^–2^	1.45 (0.27)^b^	1.65 (0.45)	−1.63 (−1.83, −1.44)
Incremental elastic modulus, mmHg	1071.62 (238.71)^b^	856.93 (131.87)	214.69 (131.2, 298.18)

Data are mean (SD).

^a^*p* < 0.05.

^b^*p* < 0.005.

### Heart-rate variability

Several time-domain measures differed between the two groups (Table 4 and Fig. 1f, g). There was moderate evidence that former AN patients had slower heart rates than the controls (adjusted mean difference −4, 95% CI −8 to 0, *p* = 0.043). There was stronger evidence of greater beat-to-beat variance in heart rate (represented by root mean square of successive RR interval differences and pRR50) among the former AN patients than the controls (adjusted mean difference 31.23 and 13.05, 95% CI 14.29 to 48.16 and 2.41 to 23.68, *p* = 0.004 and 0.017, respectively). Frequency domain measures also differed between the two groups. There was moderate evidence of a difference in total power with former AN patients exhibiting higher total power than the control group (adjusted median difference 20,992.8, 95% CI 3538.7 to 77,142.3, *p* = 0.026); indicating higher overall autonomic activity. In addition, lower LF power (adjusted mean difference −34.8, 95% CI −49.61 to 19.99, *p* < 0.001) and higher HF power (adjusted mean difference 36.18, 95% CI 21.64 to 50.73, *p* < 0.001) resulted in a lower LF-to-HF (sympathetic–parasympathetic) ratio among the former AN patients compared to the controls (adjusted mean difference −1.4601, 95% CI −2.8123 to −0.1142, *p* = 0.008) (Fig. 1h).

**Table 4:** HRV of former AN patients and controls.

	Former AN (*N* = 22)	Controls (*N* = 66)	Estimated mean/median difference (95% CI)
Time domain measures			
Average RR, ms	972.7 (114.6)^a^	913.4 (108.7)	59.4 (5.1, 113.6)
Median RR, ms	968.0 (121.2)	918.4 (114.9)	49.7 (−7.7, 107.0)
SDRR, ms	67.89 (28.85)	62.29 (22.71)	5.61 (−6.41, 17.63)
SDARR, ms	43.02 (141.70)	47.83 (24.90)	−4.82 (−41.49, 31.86)
Average heart rate, BPM	63 (7)^a^	67 (9)	−4 (−8, 0)
RMSSD, ms	79.03 (53.33)^b^	47.81 (24.89)	31.23 (14.29, 48.16)
pRR50, %	42.94 (26.91)^a^	29.89 (19.46)	13.05 (2.41, 23.68)
Frequency domain measures			
Total power, ms^2^, median (IQR)	5204.5 (2964.0–14,407.5)^a^	3623.2 (1764.4–4962.5)	20,992.8 (3538.7, 77,142.3)
VLF (0–0.04 Hz), ms^2^, median (IQR)	1048.3 (563.3–1702.5)	1265.4 (857.2–2162.5)	−137.4 (−736.9, 193.9)
LF (0.04–0.15 Hz), ms^2^, median (IQR)	1316.0 (500.3–2832.8)	928.7 (466.4–1448.1)	698.5 (−202.5, 4960.8)
LF, nu	33.81 (19.68)^b^	54.19 (14.10)	−34.80 (−49.61, −19.99)
HF (0.15–0.4 Hz), ms^2^, median (IQR)	2948.0 (1225.5–5940.0)^a^	616.5 (319.6–1245.5)	2095.5 (1019.8, 3692.8)
HF (0.15–0.4 Hz), nu	63.20 (17.80)^b^	39.61 (13.74)	36.18 (21.64, 50.73)
LF/HF	0.7202 (0.7444)^a^	1.7512 (1.2944)	−1.4601 (−2.8123, −0.1142)

Data are mean (SD) (continuous), unless specified otherwise.

*SDRR* standard deviation of RR intervals, *SDARR* standard deviation of the set of averaged RR intervals, *RMSSD* root mean square of successive RR interval differences, *pRR50* percentage of successive RR intervals that differ by more than 50 ms, *VLF* very low frequency power, *LF ms*^*2*^ absolute power of the low-frequency band, *LF nu* relative power of the low frequency band in normal units, *HF ms*^*2*^ absolute power of the high frequency band, *HF nu* relative power of the high frequency band in normal units, *LF/HF* ratio of LF-to-HF power.

^a^*p* < 0.05.

^b^*p* < 0.005.

### Endothelial function

Lastly, 46% of the former AN patients exhibited reduced endothelial function with a RHI less than the normal threshold of 1.67.^26^ The average RHI for the former AN patients was 1.59.

## Discussion

In this first study of the cardiovascular function of former AN patients, we have observed no difference in carotid IMT, but increased carotid artery stiffness, as young adults. Conversely, decreased aortic stiffness was observed. Moreover, there was persistence of high vagal tone at this early stage of adulthood, although blood pressure was normal. The former AN patients also exhibited a high prevalence of endothelial dysfunction.

There are varied reports of the effects of malnutrition on carotid IMT and stiffness. Increased carotid IMT with no difference in arterial stiffness has been observed in survivors of the Leningrad Siege seventy years on.^12^ Alternatively, individuals exposed to the Dutch Famine while in utero exhibited reduced carotid IMT^27^ but no difference in compliance.^28^ It must be noted that these famines at the time of war were different in terms of duration with the former lasting several years and the latter considered to have lasted about 6 months. Furthermore these reports are of male and female survivors, while AN predominately occurs in females.

Perhaps of more relevance to our findings are the data reported by van Abeelen and colleagues.^29^ This was a study of 7845 women from the Prospect-EPIC cohort who experienced various degrees of starvation during the Dutch famine. The risk of cardiovascular disease was stratified based on severity of exposure as well as the age of the women at the time of exposure. Overall, more extreme levels of starvation were associated with higher risk of coronary heart disease. Interestingly, this risk was highest in those women who experienced the famine between 10 and 17 years of age (corresponding with the age of eating disorder onset in our cohort). Conversely, the risk of stroke was lower in women who were exposed to famine compared to unexposed women. The authors do caution, however, that the stroke data were relatively scarce in the cohort. It is possible that stiffer carotid arteries seen in our population in the presence of a more compliant aorta may paradoxically protect against pulse propagation into the cerebral circulation and reduce the incidence of cerebrovascular accidents.

Reduced aortic stiffness, as shown by reduced pulse wave velocity, has similarly been reported in adult survivors of severe acute malnutrition in infancy.^15^ This group of survivors was a similar age to our cohort with an average age of 29 years and exhibited markedly elevated systemic vascular resistance with only slight elevation of diastolic blood pressure. The authors postulated that the higher systemic vascular resistance might be due to higher sympathetic drive and arteriolar constriction. Our population would appear to differ in having greater vagal tone but does demonstrate a high prevalence of endothelial dysfunction.

Endothelial dysfunction is a well-accepted early indicator of arterial disease and may correspond with extreme levels of starvation in the Prospect-EPIC cohort being associated with higher coronary risk.^29^ Lower RHI has been correlated with cardiovascular risk factors including obesity, dyslipidaemia, diabetes, and smoking.^30–32^ It is also an independent predictor of cardiovascular risk in patients with peripheral artery disease.^33,34^ Therefore, this finding may represent an underlying level of vascular dysfunction among the former AN patients.

The mechanisms leading to altered arterial wall properties in AN specifically are unknown. There are however studies in other populations which lend some insight. Changes in expression of multiple proteins in the extracellular matrix of the arterial wall of rat offspring after maternal undernutrition have been demonstrated.^35^ A significant decrease in the ratio of elastin–collagen was observed in both the carotid arteries and aorta, leading to stiffer vessels. Similar mechanisms could be contributing to the stiffer carotid vessels in the former AN patients, although a more compliant aorta is contradictory. While the effects of foetal malnutrition on the developing cardiovascular system are of potential interest, induced changes are unlikely to be the same as undernutrition at the stage of adolescent development. There are no histological data for arterial structure following malnutrition in childhood. Furthermore, the changes in rat offspring are dependent on sex, with males exhibiting worse adaptations.^36^ Indeed most AN patients are female. One study observed decreased aortic stiffness, but no changes in the carotid artery, in oestorgen-deficient female rats.^37^ Oestrogen deficiency is a known consequence of AN and regional differences in its modulation of vascular function could explain the contrasting carotid and aortic stiffness observed in our patients. A full metabolic workup would be required to examine this thoroughly.

The abnormal HRV among the former AN patient group demonstrates persisting changes in autonomic regulation. Parasympathetic activity (HF) was significantly greater among the former AN patients. Moreover, the low LF-to-HF ratio indicates persistence of deficient vagal inhibition when compared to the normal range of 1.5–2.0 stipulated in the HRV guidelines.^23^ Vagal hyperactivity contributes to bradycardia and hypotension in patients with AN.^4,38^ In contrast to our findings, another study in weight-recovered patients with AN reported HRV had normalised.^39^ This study was performed at early follow-up, however, with a range of 3–18 months after acute refeeding. It is possible that while BMI has returned to normal range that our patient cohort in early adulthood continues to have relatively higher levels of exercise to explain this increased vagal tone.

There were several limitations to the current study. The menstrual cycle has been shown to influence HRV and this could be particularly pertinent to our sample given the disproportionate sex distribution.^40^ There is a predominance of women in our former AN patient cohort but this reflects the sex distribution of the condition, although the number of young males affected is increasing.^41^ It is possible that males may be affected differently. In addition, there is a myriad of variables that affect cardiovascular health, such as smoking, diabetes, and hypercortisolaemia, but analysis of these was beyond the scope of this study.

The exercise history of our cohort was not clearly documented besides completion of a Commitment to Exercise Scale and a more rigorous assessment would be needed to explain our findings of increased vagal tone. It is difficult to assess whether the former AN patients were completely recovered without a detailed clinical psychological assessment. More information on the current nutritional intake of the former patients and controls, as well as fat mass and modes of exercise, would be beneficial for determining whether differences arise from persisting behaviours or a history of AN. Pre-morbid health status and any family history of cardiovascular disease would also be useful.

In addition, the former AN patients were tested after 3 h of fasting as a longer period was unethical given the high-risk participant history. This affects the accuracy of comparisons with the VICS cohort who fasted for at least 6 h prior to testing. Moreover, EndoPAT measures were unable to be compared to sex- and age-matched controls from the VICS cohort since these were not an outcome measure for that study. While this is the nature of using historical population data for comparison, it limits the significance of our findings for endothelial function.

Lastly, all patients were ascertained through specialist Eating Disorder Services and thus our findings cannot be readily generalised to populations with milder disease or those who never sought treatment. Patient historical information indicates our study sample had not experienced the greatest degree of illness chronicity or severity. A moderately, as opposed to severely or extremely, malnourished cohort and an illness duration of <1.5 years may have led to more favourable long-term outcomes. The lower prevalence of bradycardia (55%) and postural hypotension (32%) in this study population compared to data for cardiovascular complications in other AN studies^3,4,42^ also indicates lesser condition severity among our sample. It is possible that patients who fared better than others were more likely to agree to participate in research relating to their illness. Investigating the relationship between AN severity and cardiovascular impact is a potential future research direction. Furthermore, even though patients may have been recovered at the time we conducted cardiovascular assessments, any relapses in recovery or additional treatment sought could have affected our results. Inclusion of the remainder of the 36 patients who originally expressed interest could also affect the results; although chart review indicates historical characteristics (age, duration, and severity of illness) to be similar to the current sample.

In conclusion, this is the first study to conduct a long-term follow-up assessment of cardiovascular health in patients who formerly had AN during adolescence. Our findings show increased carotid artery stiffness, endothelial dysfunction, reduced aortic stiffness, and altered autonomic regulation in early adulthood. This study is preliminary in nature given the discussed limitations and further cardiovascular follow-up studies of AN, with continued monitoring into later life, should be conducted to corroborate our findings and investigate potential disease progression. Nonetheless, the persistent cardiovascular adaptations identified in our findings highlight the need for greater attention to the cardiovascular complications that occur in AN. Clinics should consider routine monitoring of AN patient’s cardiovascular health so treatments can be adapted to minimise disease risk.

## Data Availability

The data that supports the findings of this study are available from the corresponding author upon reasonable request.
